# An Efficient Starch-Based Delivery System Ameliorates Naringin’s Uptake to Mitigate Acrylamide-Induced Oxidative Stress in Mice

**DOI:** 10.3390/antiox15030384

**Published:** 2026-03-19

**Authors:** Feng Cao, Chenxing Liu, Meiyu Zheng, Yan Cao, Qile Xia, Shengmin Lu

**Affiliations:** 1 State Key Laboratory for Quality and Safety of Agro-Products, Zhejiang Key Laboratory of Intelligent Food Logistic and Processing, Institute of Food Science, Zhejiang Academy of Agricultural Sciences, Hangzhou 310021, China; caofengup@163.com (F.C.); liucx@zaas.ac.cn (C.L.); zhengmeiyu@zaas.ac.cn (M.Z.); caoyan_115@163.com (Y.C.); xiaql@zaas.ac.cn (Q.X.); 2 College of Food Science and Technology, Nanjing Agricultural University, Nanjing 210095, China

**Keywords:** naringin, octenyl succinic anhydride esterified porous starch, bioavailability, antioxidative activities, acrylamide

## Abstract

Naringin (NAR), a potent antioxidant flavonoid, suffers from low oral bioavailability, limiting its therapeutic application. Here, an efficient octenyl succinic anhydride-modified porous starch (OSAPS) carrier was adopted to enhance NAR’s delivery and efficacy against acrylamide (AA)-induced oxidative stress. In Caco-2 cells, the OSAPS-NAR complex demonstrated superior cellular uptake and more effectively mitigated AA-induced cytotoxicity compared to free NAR. Causally, this protection was attributed to its enhanced antioxidant capacity to suppress reactive oxygen species generation, maintain mitochondrial membrane potential, and prevent glutathione (GSH) depletion. Critically, these in vitro advantages translated to remarkable in vivo outcomes. The OSAPS-NAR complex led to a striking 7.91-fold increase in peak plasma concentration and an 11.05-fold increase in relative bioavailability in mice. This enhanced pharmacokinetic profile directly translated to superior antioxidant activities in serum and liver tissues by elevating total antioxidant capacity and GSH content, while simultaneously reducing malondialdehyde levels. These effects collectively led to marked amelioration of AA-induced hepatotoxicity, normalization of the liver index, and restoration of hepatic cellular architecture. In conclusion, the complex effectively overcame NAR’s pharmacokinetic limitations, thereby potentiating its ability to modulate cellular redox imbalance and protect against oxidative organ injury. This work provided a robust proof-of-concept for OSAPS-NAR as a promising nutraceutical agent for combating pathologies driven by oxidative stress.

## 1. Introduction

Naringin (NAR), a flavonoid abundant in citrus fruits and particularly in grapefruit peel [1], exhibits a wide spectrum of pharmacological activities, including antioxidant, anti-inflammatory, and anti-cancer effects [2,3,4]. However, the inherent poor aqueous solubility (0.98 mg/mL) [5] and low oral bioavailability (<5%) [6] restrict its clinical application and in vivo efficacy [7], which necessitates to be improved by an efficient carrier.

Compared with synthetic polymer or other natural carriers, starch-based carriers offer distinct core advantages, including exceptional safety and biocompatibility, abundant and accessible sources, low cost, and versatile customizability [8]. Porous starch (PS), characterized by its high specific surface area and strong adsorption capacity, is a promising carrier for drugs and nutrients [9]. However, its practical application is constrained by two major limitations: poor mechanical strength and susceptibility to digestion [10,11], as well as a limited adsorption for hydrophobic substances, such as menthol (31.83 mg/g) [12]. To address these shortcomings, octenyl succinic anhydride (OSA) esterified porous starch (OSAPS) possesses both the properties of high adsorption and amphiphilic solubilization, functioning as a more effective carrier [13]. The superiority of OSAPS has been well-documented. For instance, Li et al. [14] employed it as an emulsion-based delivery system to enhance β-carotene’s bioaccessibility. Ji et al. [12] found OSAPS with elevated degree of substitution (DS) improved menthol’s loading efficiency up to 64.34 mg/g. Our previous work also confirmed that OSAPS with elevated DS improved NAR’s payload, solubility, and sustained release [15,16], while exhibiting enhanced digestion resistance and methylene blue adsorption capacity [17]. Critically, while free NAR’s antioxidant activity is well-established, it remains unknown whether this bioactivity is preserved after its encapsulation within the OSAPS matrix.

Acrylamide (AA) is a hazardous compound that may cause neurotoxicity and carcinogenicity [18], widely found in heat processed foods rich in carbohydrates [19]. Researchers frequently utilize AA to induce oxidative stress and then evaluate a bioactive/drug’s antioxidant activity [20]. Numerous studies have demonstrated that polyphenols can significantly mitigate AA-induced oxidative stress. For instance, myricitrin (2.5–10 µg/mL) has been shown to substantially inhibit AA-induced cytotoxicity in Caco-2 cells by suppressing reactive oxygen species (ROS) production [21]. Similarly, procyanidin B2 and a cocoa polyphenolic extract mitigated AA-induced cytotoxicity in Caco-2 cells by restoring redox homeostasis and inhibiting apoptotic pathway [22]. NAR alleviated AA-induced oxidative stress in kidney tissue by enhancing its superoxide dismutase (SOD), glutathione peroxidase (GSH-Px) and catalase (CAT) activities, and GSH content while decreasing its malondialdehyde (MDA) content [23]. A similar effect was found for quercetin by lowering ROS levels while improving mitochondrial membrane potential (MMP) [24]. Although flavonoids like NAR demonstrate potent antioxidant activity in vitro, their extremely low oral bioavailability severely impedes the translation of these promising in vitro findings into in vivo tangible therapeutic effects. This gap represents a critical scientific bottleneck that demands targeted solutions. Also, it is supposed that NAR after encapsulated by PS/OSAPS would mitigate AA-induced oxidative stress due to its sustained release from carrier and the possible protective effect on its oxidative activity by the carrier, which need to be verified both in the cell and animal models with oxidative stress.

In this study, the alleviating effect of an OSAPS-NAR complex on AA-induced oxidative stress was explored both in vitro and in vivo using free NAR and a PS-NAR complex as controls. Radical scavenging capacities of the samples were assessed before and after simulated digestion. Cellular uptake of NAR from digesta was examined using a Caco-2 cell model, and the digesta’s effects on cytotoxicity, MMP, ROS and GSH levels in the AA-induced oxidative stress cells were compared. Pharmacokinetic parameters and relative bioavailability of NAR in different forms (free and encapsulated) were ascertained through a mouse model, and antioxidant indices in the serum and liver and liver pathological sections were assessed/observed in the AA-induced oxidative stress mice supplemented with samples in same NAR dose. The study would provide a theoretical basis for using OSAPS carrier to ameliorate the NAR’s bioactivity against cellular redox imbalance and hepatotoxicity.

## 2. Materials and Methods

### 2.1. Materials

Waxy corn starch was obtained from Kunlun Biochemical Co., Ltd. (Zhangye, China). Naringin (95% in purity), octenyl succinic anhydride (purity ≥ 99.5%), α-amylase (50 U/g), glucoamylase (100,000 U/g), simulated saliva fluid (SSF), simulated gastric fluid (SGF), simulated intestinal fluid (SIF), Rhodamine 123 (Rh123), detection kits of Naphthalene-2,3-dicarboxal-dehyde (NDA) content, radical (DPPH, ABTS, •O_2_^−^) scavenging activity, total antioxidant capacity (T-AOC) and antioxidative indices (SOD, GSH-Px, CAT activities, and T-AOC, GSH and MDA contents) were all obtained from Yuanye Bio-Technology Co., Ltd. (Shanghai, China). Caco-2 cells (CX0094) were purchased from BOSTER Biotechnology Engineering Co., Ltd. (Wuhan, China). Trypsin was acquired from Labgic Technology Co., Ltd. (Beijing, China). PBS buffer (0.01 mol/L, pH 7.2–7.4) was procured from Hao Yang Biological Products Technology Co., Ltd. (Tianjin, China). Penicillin-streptomycin solution, Minimum essential medium (MEM), 2′,7′-Dichlorofluorescin diacetate (DCFH-DA), and MMP test kit were sourced from Solarbio Science & Technology Co., Ltd. (Beijing, China). Fetal bovine serum (FBS) was obtained from Tianhang Biotechnology Co., Ltd. (Huzhou, China). Cell counting kit-8 (CCK-8) was purchased from Beyotime Biotechnology Co., Ltd. (Shanghai, China). Acrylamide was received from Macklin Biochemical Technology Co., Ltd. (Shanghai, China). SPF grade male KM mouse weighing 25 ± 3 g was purchased from Qizhen Laboratory Animal Technology Co., Ltd. (Hangzhou, China). Hematoxylin and eosin (HE) were acquired from Beso Biotechnology Co., Ltd. (Zhuhai, China). Unless otherwise specified, all chemicals were of analytical grade.

### 2.2. Preparation of PS/OSAPS-NAR Complexes

PS and OSAPS were prepared according to the reference [15]. Briefly, PS was produced via enzymatic hydrolysis to waxy corn starch using a mixture of α-amylase and glucoamylase at a 6:1 mass ratio (*w*/*w*). Subsequently, OSAPS was synthesized by esterifying PS with OSA at a concentration of 7% (based on the dry weight of PS) under alkaline conditions (pH 8.5). The DS of the resulting OSAPS was determined to be 0.0427 by titration. The DS value indicates the extent of starch modification by OSA, i.e., the degree of starch esterification increases with elevated DS value. The OSAPS has high loading efficiency and capacity for NAR, with a good sustained-release effect on NAR [15].

PS/OSAPS-NAR complexes were prepared using a modified ultrasonic method [25]. Briefly, a 0.22% (*w*/*w*) NAR ethanol solution and a 5 mg/mL aqueous starch slurry (1:2 in starch-to-NAR mass ratio) were individually sonicated (500 W, 10 min). The NAR solution was added dropwise to the starch slurry at 50 °C under stirring (500 rpm) until the ethanol evaporated. The mixture was further sonicated (100 W, 30 min), centrifuged (10,000 rpm, 30 min), and freeze-dried to obtain the final powder. Unbound NAR in the supernatant was quantified via HPLC [15]. The loading efficiency (*LE*) and loading capacity (*LC*) of NAR were calculated using Equations (1) and (2), respectively.

(1) $$LE\;(\%)=\frac{Total\;amount\;of\;naringin\;(g)\;used-Free\;naringin\;(g)\;in\;supernatant}{Total\;amount\;of\;naringin\;(g)\;used}\;\times \;100$$

(2) $$LC\;(\%)=\frac{Total\;amount\;of\;naringin\;(g)\;used\;-\;Free\;naringin\;(g)\;in\;supernatant}{Total\;amount\;of\;naringin\;and\;starch\;(g)\;used}\;\times \;100$$

### 2.3. In Vitro Digestion

Samples of free NAR and the PS/OSAPS-NAR complex with equivalent mass (10 mg) of NAR were put in separate flasks. The sequential digestion was initiated by adding 5 mL of SSF. After 5 min of incubation, 50 mL of SGF was introduced. Two hours later, 25 mL of SIF was added to the mixture to commence the intestinal phase. The entire digestion process was conducted at 37 °C in a shaking water bath (100 rpm) under dark conditions.

### 2.4. Determination of Antioxidant Activities

The antioxidant activities of the samples (both pre- and post-digestion) were quantified using the kits in strict accordance with the manufacturer’s instructions. Briefly, the DPPH and ABTS free radical scavenging capacity was determined by spectrophotometry at a wavelength of 517 nm and 734 nm using Trolox as standard, while the O_2_^−^ radical scavenging capacity was measured using the resorcinol method at 320 nm, and expressed as relative percentage.

### 2.5. Cell Experiments

#### 2.5.1. Cell Culture

Caco-2 cells were cultured in 95% MEM supplemented with 1% penicillin-streptomycin solution and 5% FBS in an atmosphere of 5% CO_2_ at 37 °C. Cell passage was performed approximately every two days. When the cell saturation density in the culture bottle exceeded 80%, trypsin was employed to digest the cells, and the medium was replaced to ensure normal cell growth. During culture, cell observation was conducted under a microscope (MD-4, NARISHIGE, Setagaya, Japan). The whole operation process of the cell experiment was carried out under an ultra-clean environment.

#### 2.5.2. Determination of Cell Viability

Cell viability was evaluated following the protocol described by the report [26], with minor modifications. Briefly, cells were seeded into 96-well plates at a density of 5 × 10^3^ cells/well. After 24 h of incubation, the culture medium was removed, and the wells were washed once with the PBS before subsequent treatments. Cell culture in the CK was added with 100 μL of medium, whereas those in the sample groups were added with 100 μL of free NAR or PS/OSAPS-NAR complex digesta with different NAR concentrations (10, 15, 20, 25, 30 μg/mL NAR). Cell viability was determined using the CCK-8 assay kit and expressed as the percentage of absorbance in the sample group to that in CK.

#### 2.5.3. Cellular Uptake Study

Cell uptake was studied according to the previous report [26]. Caco-2 cells were seeded into 6-well plates at a density of 2 × 10^5^ cells/well and cultured for 24 h. The medium was then aspirated, and the cell monolayers were washed twice with the PBS (2 mL). Subsequently, the cells were incubated for 3 h with culture medium containing digesta (prepared as 2.3) of either free NAR or the PS/OSAPS-NAR complex (containing 15 μg/mL NAR) treated with Rh123 (5 μmol/L). After incubation, the treatment solutions were removed, and the cells were washed three times with the PBS. Cellular green fluorescence was observed and captured using a stereo fluorescence microscope (M165FC, Leica, Wetzlar, Germany), whose intensity was quantified using ImageJ 1.53t software (National Institutes of Health, Bethesda, MD, USA).

#### 2.5.4. Determination of Cellular Reactive Oxygen Species Levels

The ROS levels were measured using the DCFH-DA assay, following a reported method [20], with minor modifications. Briefly, cell culture and treatment with digesta were same as Section 2.5.3. After removing the treatment solutions with a pipette gun, ROS production was induced by incubating the cells with 1 mL of 5 mM AA in PBS per well for 24 h. The cells were then washed twice with the PBS and incubated with 1 mL of 10 µM DCFH-DA for 30 min, with gentle agitation every 5 min to ensure uniform probe uptake. Finally, after a final wash with the PBS to remove the extracellular probe, the intracellular green fluorescence from DCF was observed with the stereo fluorescence microscope. The mean fluorescence intensity was quantified using the ImageJ software.

#### 2.5.5. Determination of Cellular Mitochondrial Membrane Potential

The MMP was determined using the fluorescent probe JC-1 [27]. Briefly, cell culture and treatment with the digesta were the same as Section 2.5.3. The cells were then incubated with 0.75 mL of complete medium and 0.25 mL of JC-1 dyeing solution for 20 min. Following incubation, the supernatant was aspirated, and the cells were washed twice with ice-cold JC-1 dyeing buffer (1×). Finally, 1 mL of fresh culture medium was added to each well, and the cells’ fluorescence images were immediately acquired using the stereo fluorescence microscope. The fluorescence intensities of red (J-aggregates) and green (J-monomers) were quantified using the ImageJ software. The MMP level was expressed as the ratio of red to green fluorescence intensity.

#### 2.5.6. Determination of Cellular Glutathione Level

Cell culture and treatment with the digesta were the same as Section 2.5.3. Each well of cells in the sample treated groups was incubated with 1 mL of 50 μM NDA for 30 min. After incubation, the culture medium was aspirated, and the cells were washed three times with the PBS to remove any non-internalized NDA. The green fluorescence images of the cells were then acquired using the stereo fluorescence microscope. Finally, the mean fluorescence intensity of NDA was quantified using the ImageJ software.

### 2.6. Adaptive Feeding for Mice

The animal experiments complying with the ARRIVE guidelines were conducted under approval (YXSW2306210537) from Committee of Ethics and Welfare for Experimental Animals, Zhejiang Academy of Agricultural Sciences. The mice were normally fed under the conditions of 20–26 °C, 40–70% relative humidity, and a 12 h light–dark alternation. All mice were acclimatized to the laboratory conditions for 5 days with ad libitum access to standard feed and water. Following the acclimatization period, the animals were randomly assigned to experimental groups.

### 2.7. Pharmacokinetic Experiments

#### 2.7.1. Group Administration and Sampling of Mice

The experimental group administration and sampling of mice were designed using a literature-described approach [28,29]. Fifteen mice were randomly divided into three groups (5 per group): NAR, PS-NAR complex, and OSAPS-NAR complex. Before the experiment, all animals were fasted for 12 h with free access to water. At the start of the experiment, each mouse’s body weight was monitored, and the dosage of the reagent administered by gavage was also adjusted accordingly. Mice in each group were administered at a 50 mg/kg NAR equivalent dose (0.1 mL/10 g bw). At designated time point (0.083, 0.25, 0.5, 1, 2, 4, 6, 8, 12, and 24 h) of post-administration, the blood sample (50 μL) was collected from the orbital venous bundle of each mouse and put into a 1.5 mL microcentrifuge tube containing heparin sodium anticoagulant. The sample was immediately centrifuged at 3000 r/min for 10 min at 4 °C to separate the plasma. The resulting plasma supernatant was carefully collected, transferred to a new tube, and stored at −80 °C until analysis.

#### 2.7.2. Determination of Naringin Concentration in Mice Plasma

The extraction of NAR in mouse plasma was in compliance with report of Liu et al. [30]. The combined supernatants were evaporated to dryness under a nitrogen stream at 40 °C, redissolved in 100 μL of methanol by vortex for 2 min and filtered through a 0.22 μm organic filter membrane before a 10 μL aliquot of the filtrate injected into a HPLC system for NAR quantification, with the determination method set as described [31]. The ratio of mobile phase, flow rate, sample size, column temperature and detection wavelength were 35:65 (0.2% acetic acid water: methanol, *v*/*v*), 1 mL/min, 10 μL, 25 °C, and 283 nm, respectively.

#### 2.7.3. Pharmacokinetic Parameter Calculation

The pharmacokinetic parameters were calculated using the Drug Absorption System (DAS) 2.0 software (GastroPlus^®^, Simulations Plus, Inc., Durham, CA, USA), including the peak concentration *C_max_*, peak time *T_max_*, distribution half-life *T*_1/2*α*_, elimination half-life *T*_1/2*β*_, area under the curve *AUC*_0–*t*_ and *AUC*_0–*∞*_, and mean residence time *MRT*_0–*∞*_.

#### 2.7.4. Relative Bioavailability Calculation

Relative bioavailability was calculated according to the following Equation (3),

(3) $$\mathrm{Relative}\;\mathrm{bioavailability}\;(\%)=\frac{{AUC}_{T}}{{AUC}_{R}}\times \frac{D_{R}}{D_{T}}\;\times \;100\;$$

where *AUC_T_* and *AUC_R_* are the *AUC*_0–*t*_ of the test and reference preparations for intragastric administration, respectively, and *D_R_* and *D_T_* are the doses of the reference and test reagents [32], referring to free NAR and PS/OSAPS-NAR, respectively.

### 2.8. In Vivo Antioxidant Experiments

#### 2.8.1. Grouping and Administration of Mice

Experimental grouping and administration of mice were designed with the methods reported in the literature [24,33]. Twenty-five mice were divided into five groups. The normal (CK) mice were given continuous intragastric administration of physiological saline at a dose of 0.1 mL/10 g bw/day for 7 days. Mice in AA group were administered with AA via intraperitoneal injection at a dose of 50 mg/kg bw/day and solution (dissolved in physiological saline) volume of 0.05 mL/10 g bw/day for 7 days. For free NAR and AA combined treatment group, NAR was intragastric administered to mice at 100 mg/kg bw/day and a solution volume of 0.1 mL/10 g bw/day for 7 days; meanwhile, the mice were injected with AA for 7 days at the same dose and solution volume as those in AA group. For PS/OSAPS-NAR complex and AA combined treatment group, the mice were given PS/OSAPS-NAR complex and AA with the same dose and solution volume as those in free NAR and AA combined group for 7 days. All reagents were freshly prepared immediately prior to administration. Twenty-four hours after the final administration, the mice were fasted overnight and then sacrificed by cervical dislocation.

#### 2.8.2. Determination of Body Weights and Liver Indices of Mice

Each mouse’s body weight was recorded daily at a consistent time. Immediately after sacrificed, livers were excised, rinsed with ice-cold physiological saline, blotted dry with filter paper, and weighed. The liver index was then calculated as a ratio of liver weight to the final body weight [34]. The livers were then quick-frozen in liquid nitrogen and kept in a refrigerator at −80 °C for subsequent use.

#### 2.8.3. Determination of Antioxidant Indices in Serum and Liver

Antioxidant indices in serum and liver were determined following the procedures published in the literature [23]. At the conclusion of the experiment, blood sample (~0.8 mL) was collected from each mouse via the retro-orbital sinus, allowed to clot by incubation at 37 °C for 1 h and 4 °C overnight for retraction. Serum was then separated by centrifugation at 3000 r/min for 10 min at 4 °C, collected and stored at −80 °C until analysis. The liver was accurately weighed (50 mg), cut into pieces before being mixed with 9 times the volume (mass/volume) of pre-cooled physiological saline and homogenized. The homogenate was centrifuged at 3000 r/min for 10 min at 4 °C to collect the supernatant stored at −80 °C until analysis. The activities of SOD, GSH-Px, and CAT, and the contents of T-AOC, GSH, and MDA in serum and liver were determined using commercial kits. The assays were performed as follows: SOD (NBT-riboflavin method, 560 nm), GSH-Px (422 nm), CAT (ammonium molybdate method, 405 nm), T-AOC (ABTS method, 734 nm), GSH (DTNB method, 412 nm) and MDA (TBA method, 535 nm).

#### 2.8.4. Pathological Observation of Liver Tissue

Observation of liver pathological sections was slightly modified with the reference [24]. The liver tissues were fixed in 4% paraformaldehyde for 24 h, dehydrated through a graded series of ethanol, cleared in xylene, embedded in paraffin wax, sectioned into slices in 3–4 μm thickness, mounted on glass slides and baked at 65 °C. After deparaffinization, the sections were stained with H&E, dehydrated, cover slipped, and digitized using a film scanner (Pannoramic MIDI model, 3D HISTECH company, Budapest, Hungary) for analysis.

### 2.9. Statistical Analysis

All experimental data are presented as mean ± standard deviation (SD). Statistical analysis was performed using SPSS 21.0 (IBM, New York, NY, USA) and Origin 2021b (Origin Lab Corporation, Northampton, MA, USA). One-way analysis of variance (ANOVA) followed by Duncan’s multiple range test was used to determine significant differences between group means, with *p* < 0.05 considered statistically significant. The DAS was used to calculate pharmacokinetic parameters, and Slide Viewer (3DHISTECH Ltd., Budapest, Hungary) was employed to analyze pathological liver sections.

## 3. Results and Discussion

### 3.1. In Vitro Antioxidant Activity

**Figure 1:**
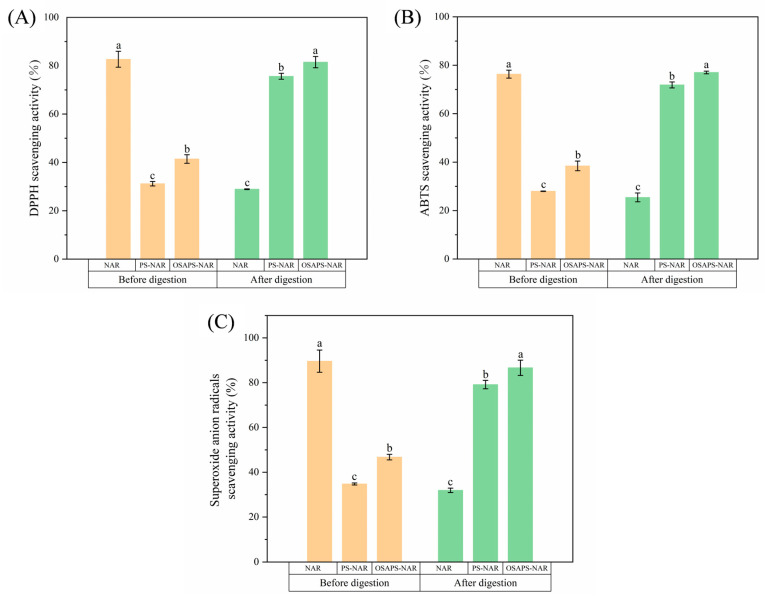
Antioxidant activities of free NAR and PS/OSAPS-NAR complexes before and after digestion. (**A**) DPPH scavenging ability; (**B**) ABTS scavenging ability; (**C**) Superoxide anion radicals scavenging ability. Values are means ± SD (n = 3) and different letters indicate significant difference (*p* < 0.05). NAR: naringin; PS-NAR: porous starch–naringin complex; OSAPS-NAR: octenyl succinic anhydride modified porous starch–naringin complex (Same abbreviations for these samples in the following figures and table).

The determinations of DPPH, ABTS and superoxide anions scavenging abilities are frequently employed to assess the antioxidant activity of a candidate substance due to their simple operation, short time, and favorable results. It was discovered that the free NAR had significantly lower antioxidant activity (*p* < 0.05) after digestion (Figure 1), suggesting that free NAR be partly destroyed during digestion and need to be protected by a carrier. The *LE* and *LC* of PS on NAR were 71.68% and 23.89%, respectively. PS could adsorb NAR because its surface had a significant number of holes and had a large specific surface area and pore volume [17]. However, OSAPS had higher *LE* and *LC* to NAR compared to PS, reaching up to 86.85% and 28.95%, respectively. This suggested that the esterification reaction further improved the adsorption performance of PS on NAR. The specific surface area of PS rose after esterification, while the carboxyl group on OSA increased the number of adsorption sites for OSAPS, thus increasing its *LE* and *LC* to NAR [17].

Figure 1 revealed that starch–naringin complexes had significantly lower antioxidant activity (DPPH, ABTS and •O_2_^−^ scavenging activities) compared with the free NAR prior to digestion (*p* < 0.05). This was because the complexes shielded NAR during the antioxidant activity detection, weakening its interaction with free radicals [26]. However, the undigested OSAPS-NAR complex exhibited stronger antioxidant activity than the PS-NAR complex (*p* < 0.05), probably due to OSAPS’ higher loading rate on NAR. Notably, the starch–naringin complexes in the digestive solutions had considerably higher antioxidant activities than the free NAR digesta (*p* < 0.05), confirming the protective effects of the starch carriers due to their sustained-release effect on NAR during digestion. Worthwhile, OSAPS-NAR digesta had higher antioxidant activity than PS-NAR digesta (*p* < 0.05), attributed to OSAPS’ higher NAR loading rate and better protective effect on NAR due to its amphipathy and more resistance to digestion [15].

### 3.2. Cell Viability and Cytotoxicity

**Figure 2:**
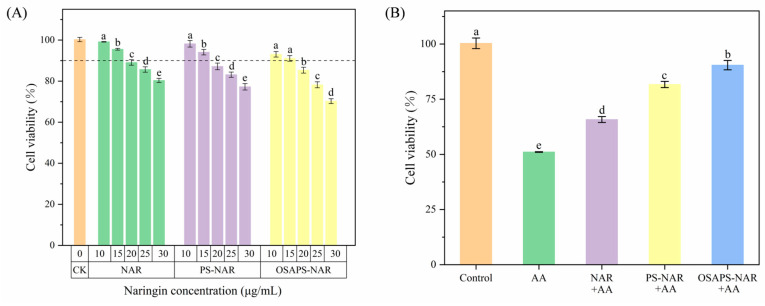
Effects of digesta of NAR in different forms without (**A**) and with AA (**B**) on viability of Caco-2 cells. CK: the control; AA: acrylamide. Values are means ± SD (n = 3) in the figure; different letters indicate significant difference (*p* < 0.05).

Figure 2A exhibits how varied NAR concentrations in the free NAR and PS/OSAPS-NAR complexes digesta affect the viability of Caco-2 cells. Compared to the CK, as the concentrations of NAR in the samples increased, the cell viability decreased progressively. The cell viability in all three sample-treated groups exceeded 90% when their NAR concentration was less than 15 μg/mL, indicating that the 0–15 μg/mL NAR in digesta had no influence on cell growth. Therefore, 15 μg/mL NAR contained in the samples was selected as the treating concentration for subsequent experiments. Noteworthily, the slight decline in viability of the OSAPS-NAR group might be caused by over-released OSA from the carrier, which awaits to be explored on its effect on cell viability and safety in animal models.

Figure 2B showed that AA treatment alone significantly reduced cell viability (51.08%) compared to the CK (*p* < 0.05). However, AA combined with NAR samples treatment significantly enhanced cell viability, with the starch–naringin complexes digesta significantly outperforming the free NAR digesta (*p* < 0.05). The cell viability increased to 90.45% after being treated with the OSAPS-NAR complex digesta plus AA. This phenomenon might be attributed to the fact that the OSAPS-NAR complex digesta has a relatively high antioxidant activity which is linked to a higher inhibitory effect on cytotoxicity [22].

### 3.3. Cell Uptake

**Figure 3:**
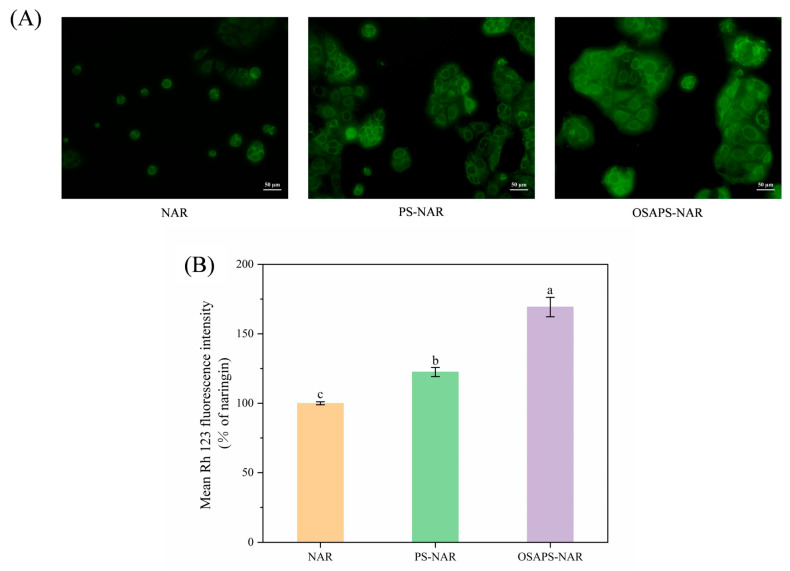
Effects of different sample digesta treatments on NAR uptake in Caco-2 cells. (**A**) Fluorescence signal images; (**B**) mean fluorescence intensity. Rh 123: rhodamine 123. Values are means ± standard deviation (n = 3) in the figure; different letters indicate significant difference (*p* < 0.05).

Live cell microscopy revealed a clear hierarchy in green fluorescence intensity among the treatment groups (Figure 3A). Specifically, after digestion and incubation with the cell, the OSAPS-NAR complex group yielded the most intense signal, followed by the PS-NAR complex, and both exhibiting substantially stronger fluorescence than the free NAR group. The quantitative analysis of Rh123 fluorescence (Figure 3B) demonstrated a significant difference among the treatment groups (*p* < 0.05). Specifically, the OSAPS-NAR complex group yielded the highest fluorescence intensity (169.26%), followed by the PS-NAR complex group (122.50%). The increased fluorescence intensity suggests that the complexes’ digesta may enhance NAR uptake by cells, with OSAPS-NAR complex having better effect.

### 3.4. Cellular ROS Level, MMP and Glutathione Depletion

**Figure 4:**
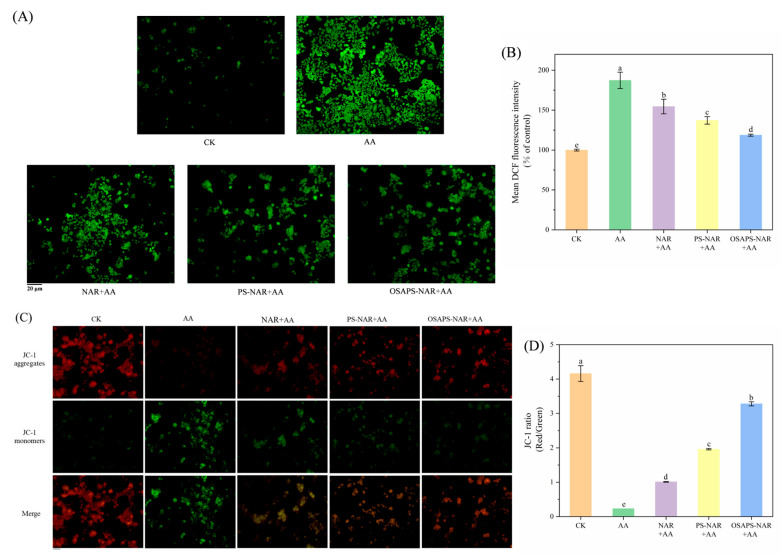

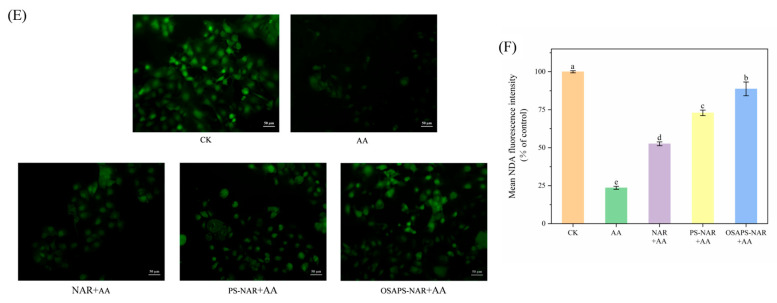
Effects of different sample digesta treatments on cell reactive oxygen species levels, mitochondrial membrane potential and glutathione depletion. (**A**) DCF fluorescence signal image; (**B**) mean DCF fluorescence intensity; (**C**) JC-1 fluorescence image; (**D**) red/green JC-1 fluorescence intensity ratio. (**E**) NDA fluorescence signal images; (**F**) mean NDA fluorescence intensity. Caco-2 cells (10^5^ cells/mL, 1 mL) treated with different digesta samples plus AA (5 mM, 1 mL) for 24 h were used to determine ROS levels (**A**,**B**), MMP (**C**,**D**) and glutathione depletion (**E**,**F**). Values are means ± standard deviation (n = 3) in the figure; different letters indicate significant difference (*p* < 0.05).

According to the results of the above antioxidant activity study, digested free NAR and starch–naringin complexes displayed strong antioxidant activity, suggesting that they might also inhibit AA-induced ROS overproduction in cells. As shown in Figure 4A,B, the cells after being exposed to AA had significantly higher mean fluorescence intensity (187.32%) compared to the control (*p* < 0.05). However, the free NAR and starch–naringin complexes treatments (after digestion and incubation) resulted in significantly lower average fluorescence intensity in the cells than the AA treatment (*p* < 0.05), with the lowest (118.71%) in the OSAPS-NAR complex group. Thus, both treatments of NAR and its two complexes digesta could considerably suppress AA-induced intracellular ROS generation, with the OSAPS-NAR complex digesta being the most effective due to its high uptake for NAR in cells. In conclusion, NAR protected cells against AA-induced oxidative damage by inhibiting intracellular ROS generation and restoring cellular redox balance, with that from OSAPS-NAR complex exhibiting best protection against oxidative damage due to its high antioxidant activities (Figure 1) and sustained release effect during digestion [15].

As shown in Figure 4C, the CK cells’ merging fluorescence image primarily exhibited red signals, whereas the AA-treated mainly showed green ones, suggesting a sharp decline in MMP, i.e., mitochondrial dysfunction, consistent with previous studies [35]. However, cells treated with the free NAR or complexes’ digesta plus AA displayed an obvious increase in red fluorescence and a visible reduction in green signals, indicating that MMP’s cells were normalizing. Figure 4D depicts the variations in MMP of the cells after different treatments (*p* < 0.05). AA treatment resulted in the lowest JC-1 ratio, indicating severely impaired mitochondrial function in the cells. However, pretreatment with free NAR or PS/OSAPS-NAR complex digesta considerably improved the JC-1 ratio in the AA-treated cells (*p* < 0.05), with the OSAPS-NAR pretreatment showing highest (3.28). The recovery in MMP of AA-treated cells by NAR and PS/OSAPS-NAR complex digesta is attributed to their ROS scavenging ability since the generation of ROS in cells is associated with the breakdown of MMP, a mitochondrial dysfunction when cells are exposed to AA [36].

AA treatment led to a significant depletion of intracellular GSH, reducing its level to 23.59% of that in the CK, while the free NAR or PS/OSAPS-NAR complex (after digestion) supplement significantly reduced the GSH depletion caused by AA (*p* < 0.05) as the fluorescence intensity of NDA in cells increased to 52.57%, 72.93%, and 88.70%, respectively (Figure 4E,F). Among them, the OSAPS-NAR complex treatment was the most effective in alleviating AA-induced GSH consumption. In summary, NAR supplement could modulate oxidative damage induced by AA through the modulation of the GSH antioxidant system. Notably, this protective effect was most pronounced when delivered as the OSAPS-NAR complex due to its potent antioxidative potency.

### 3.5. Pharmacokinetics and Relative Bioavailability of NAR

**Figure 5:**
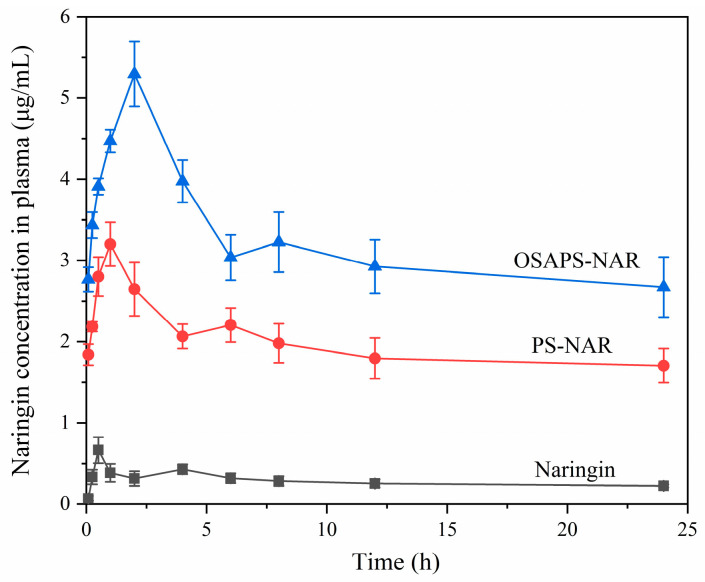
Plasma NAR concentration–time curves of the samples after intragastrically administrated in mice (n = 5).

Figure 5 depicts the drug–time curve for mice following intragastric administration of free NAR or the PS/OSAPS-NAR complex. The double-peak phenomena in the sample groups might be associated with the enterohepatic circulation and reabsorption of NAR in vivo, suggesting that NAR’s pharmacokinetics be affected by its presence form [36]. Pharmacokinetic analysis indicated the two-compartment model best matched all samples’ drug–time curves in this experiment, and the particular parameters were provided in Table 1. The C_max_ differed significantly among the three groups (*p* < 0.05), with highest in OSAPS-NAR complex group (1.66- and 7.91-fold that in the PS-NAR complex and free NAR groups). This indicated that NAR in the OSAPS-NAR complex was most readily absorbed within mice body. In addition, the OSAPS-NAR complex supplement resulted in the highest values of T_max_, T_1/2α_, and T_1/2β_, suggesting that NAR from the complex stay in gastrointestinal tract for a longer period than those from the other forms, with more weakened metabolism and elimination, resulting in more complete absorption. Also, AUC_0-T_ and AUC_0-∞_ of NAR in this complex treated group were the largest, with 10.97- and 37.03-fold those in the free NAR treated group. These findings revealed that the OSAPS-NAR complex supplement had the best efficiency in promoting NAR absorption. Furthermore, the MRT_0-∞_ was significantly prolonged in the groups treated with starch-naringin complexes compared to that in the free NAR group, indicating that the carriers efficiently increased NAR’s transit time in the body, hence prolonging the recycling time of NAR in the blood.

Table 1 also displayed that the PS/OSAPS-NAR complex increased NAR’s relative bioavailability by 6.90 times and 11.05 times, respectively, which could also enhance its in vivo bioefficacy. The in vitro digestion results previously studied by our team can explain this phenomenon. Compared to PS-NAR complex, the OSAPS-NAR complex with higher DS could protect more NAR from being destroyed by stomach acid, and its anti-digestibility rendered by OSA could delay NAR release from the carrier and prolong its residence time in the body [17].

### 3.6. Body Weights and Serum Antioxidant Indices

**Figure 6:**
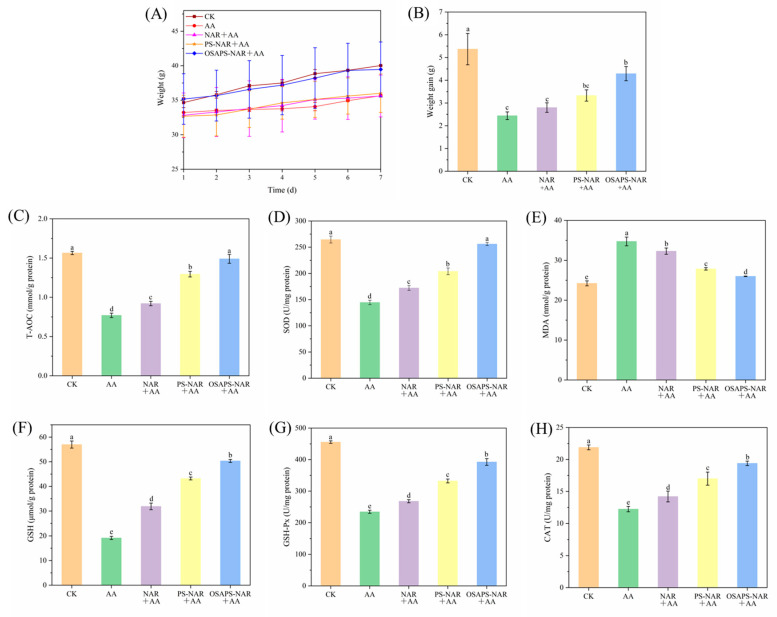
Effects of different sample treatments on body weight (**A**), weight gain (**B**) and serum antioxidant indices (**C**–**H**) of mice. T-AOC: total antioxidant capacity; SOD: superoxide dismutase; MDA: malondialdehyde; GSH: glutathione; GSH-Px: glutathione peroxidase; CAT: catalase. Values are means ± standard deviation (n = 5) in the figure; different letters indicate significant difference (*p* < 0.05).

Figure 6A revealed that the body weights of mice in the CK and the OSAPS-NAR complex plus AA-treated groups grew normally, whereas those of the other groups increased somewhat slower, with the slowest in the AA-treated alone. As seen from the body weight increment column charts (Figure 6B), compared with the CK (5.27 g), the other groups showed significant reductions (*p* < 0.05), with the least in the AA group (2.44 g), indicating that the toxicity caused by AA impaired mice’s normal growth. However, weight gains of the NAR in different forms plus AA-treated groups were increased, which were 2.80, 3.33 and 4.29 g, indicating that NAR supplement could help recover mice’s growth damaged by oxidative stress, with the OSAPS-NAR complex treatment having the best effect, as shown in its weight gain most approaching that of the CK.

As shown in Figure 6C–H, compared with the CK, the AA-treated mice had significantly decreased T-AOC and GSH content, and SOD, GSH-PX and CAT activities, while significantly increased levels of MDA, a product of lipid peroxidation in their serum (*p* < 0.05). The free NAR and the PS/OSAPS-NAR complex supplements significantly ameliorated all antioxidant indices of serum in the mice with AA-induced oxidative stress (*p* < 0.05), indicating that NAR significantly improved the antioxidant capacity and reduced the cell oxidative damage caused by AA exposure. NAR helped to restore the antioxidant parameters of the mice to normal levels, with the OSAPS-NAR complex having the most prominent effect among the three NAR existing forms (*p* < 0.05).

### 3.7. Liver Indices, Antioxidant Indices and Pathological Observation

**Figure 7:**
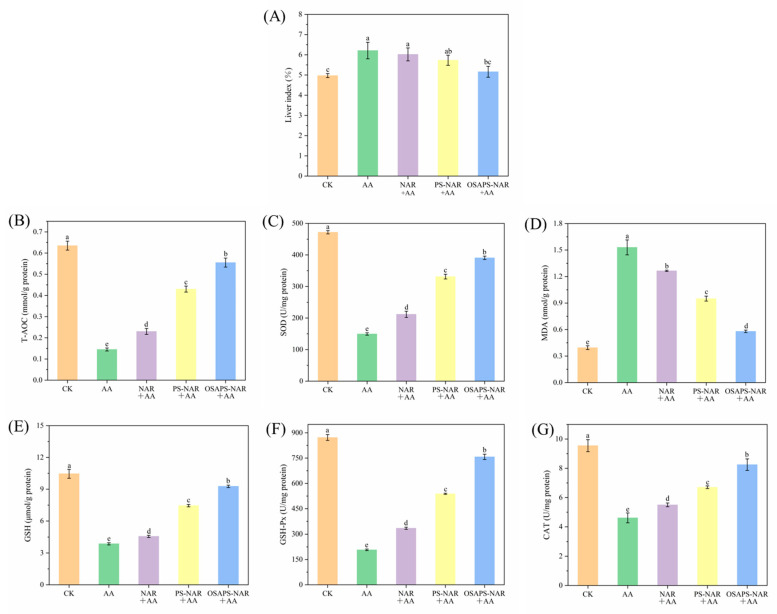

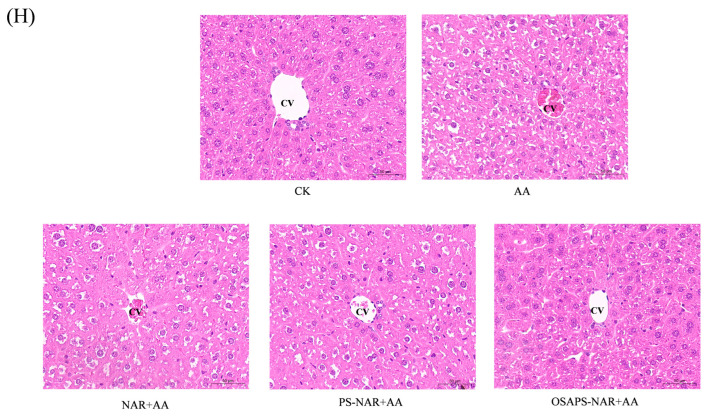
Effects of different sample treatments on liver indices (**A**), antioxidant indices (**B**–**G**) and pathological sections (**H**) of mice in AA-induced oxidative stress. CV: central vein. Values are means ± standard deviation (n = 5) in the figure; different letters indicate significant difference (*p* < 0.05).

Figure 7A depicts the liver indices change in mice, which indirectly reflected the extent of liver damage induced by oxidative stress and the alleviating effects by NAR in different forms. AA treatment resulted in a significantly higher liver index (6.21%) in the mice compared to the CK (4.97%) (*p* < 0.05). However, the free NAR and PS/OSAPS-NAR complex treatments led to a decrease in the liver indices of AA-treated mice, indicating that NAR could alleviate the toxic damage produced by AA to some extent. Nonetheless, there was no significant difference in liver indices among the free NAR + AA, PS-NAR + AA and AA-treated groups, suggesting that the free NAR/PS-NAR supplement had a weak alleviating effect on the oxidative stress. In contrast, the OSAPS-NAR + AA group exhibited a significantly lower liver index than the AA group (*p* < 0.05), reaching a level comparable to that in the CK (*p* > 0.05), due to the complex’s higher antioxidant activity and relative bioavailability. These results demonstrate that the OSAPS-NAR complex confers significant protection against AA-induced oxidative damage in mice.

Compared to the CK, the AA-induced model mice exhibited significant decreases in hepatic antioxidant indices and increase in MDA content (*p* < 0.05) (Figure 7D). Whereas after simultaneously supplemented with the free NAR or PS-/OSAPS-NAR complex, the antioxidant indices (Figure 7B,C,E–G) in the AA-treated mice were significantly enhanced in an order of OSAPS-NAR complex followed by PS-NAR complex and free NAR treatments (*p* < 0.05), consistent with their relative bioavailability order. In conclusion, AA induced oxidative stress damaged antioxidant capacity of mice’s livers, while NAR could reduce this toxic damage to some extent by eliminating the free radicals, with the OSAPS-NAR complex having the best effect on recovering the antioxidant capacity.

As shown in Figure 7H, liver cells in the CK were orderly arranged around the central vein in a radiative manner, with clear cell structure, normal nuclei, and hepatic cords separated by hepatic sinuses. However, the livers of mice in the AA group showed obvious pathological changes, including severe central venous congestion, disordered arrangement of hepatocytes, unclear polygonal edges of hepatocytes, vacuoles of different sizes with lipid droplets in the cytoplasm, cell nuclei squeezed to one side of the cells, obvious expansion of hepatic blood sinuses, and disordered hepatic cords. Similar lesions were observed in mice hepatocytes treated with AA [24]. Furthermore, it was revealed that the pathological degree of liver sections of AA-treated mice was obviously reduced after being simultaneously treated with free NAR or starch–naringin complex. Because of its lowest relative bioavailability, free NAR supplement had the weakest improvement effect on metastatic hepatocytes, resulting in partial congestion in the central vein and more lipid vacuoles. Comparatively, the protective effect on liver tissue by PS-NAR complex supplement was significantly enhanced due to its relative bioavailability being prominently improved to 689.90%, as exhibited in slightly congested central vein and no significantly expanded hepatic blood sinuses. Notably, the structure of hepatocytes in mice treated with the OSAPS-NAR complex plus AA was almost no congestion in the central vein and had minimal number of intracellular vacuoles, which was close to the normal cells. This significant amelioration was probably due to the higher relative bioavailability (1105.30%) of NAR rendered by the OSAPS-NAR complex, 1.60 times that by the PS-NAR complex (Table 1). In summary, NAR could protect liver cells and reduce their damage from AA toxicity, and the best protective effect on liver tissue by the OSAPS-NAR complex was probably due to its highest relative bioavailability for NAR and highest antioxidant activity.

## 4. Conclusions

Herein, we demonstrated that an efficient delivery carrier significantly enhanced NAR’ digestion stability, bioavailability, and antioxidant efficacy. NAR’s antioxidant activity decreased after digestion; however, after being complexed by PS/OSAPS, its DPPH, ABTS and •O_2_^−^ scavenging activities remain high in digesta. The OSAPS-NAR complex digesta facilitated superior NAR’s cellular uptake in Caco-2 cells, and consequently exhibited enhanced potency in mitigating AA-induced oxidative stress, as evidenced by potently suppressing ROS accumulation, stabilizing MMP and preventing GSH depletion. Critically, these in vitro advantages translated to significant in vivo therapeutic benefits, as exhibiting markedly improved NAR’ oral bioavailability, superior systemic and hepatic antioxidant capacity and profound attenuation of AA-induced liver injury in a murine model.

Collectively, our findings established that the OSAPS-NAR complex effectively overcame free NAR’s inherent limitations, maximizing its ability to modulate cellular redox homeostasis. This work would provide preliminary evidence for the OSAPS-NAR complex as a promising nutraceutical agent for alleviating pathologies underpinned by oxidative stress. However, in future study, it is essential and critical to reveal OSAPS-NAR digesta’ cellular uptake mechanism and its function on gene expression and metabolic pathways related with cell growth, injury and even apoptosis provoked by oxidative stress. Clinical investigations such as assessing the complex’s safety, tolerability and efficacy in healthy/elder volunteers are also warranted to translate this promising strategy into human health applications.

## Figures and Tables

**Table 1:** Pharmacokinetic parameters and relative bioavailability of NAR in free NAR and PS/OSAPS-NAR complexes after intragastrically administrated in mice (n = 5). Different letters in the same row indicate significant difference between different treatment groups (*p* < 0.05).

Pharmacokinetic Parameters	NAR	PS-NAR Complex	OSAPS-NAR Complex
C_max_ (μg/mL)	0.67 ± 0.16 ^c^	3.20 ± 0.09 ^b^	5.30 ± 0.40 ^a^
T_max_ (h)	0.50	1.00	2.00
T_1/2α_ (h)	0.17 ± 0.11 ^c^	0.64 ± 0.02 ^b^	1.14 ± 0.02 ^a^
T_1/2β_ (h)	32.18 ± 4.71 ^b^	65.93 ± 4.79 ^a^	69.32 ± 0.00 ^a^
AUC_0–t_ (mg/L·h)	6.94 ± 1.23 ^c^	47.57 ± 5.30 ^b^	76.14 ± 7.66 ^a^
AUC_0–∞_ (mg/L·h)	12.86 ± 2.20 ^b^	189.37 ± 115.36 ^ab^	476.12 ± 196.59 ^a^
MRT_0-∞_ (h)	34.67 ± 2.05 ^c^	84.44 ± 47.77 ^b^	142.70 ± 49.91 ^a^
Relative bioavailability (%)	—	689.90 ± 46.09 ^b^	1105.30 ± 85.79 ^a^

## Data Availability

The original contributions presented in this study are included in the article. Further inquiries can be directed to the corresponding author.
